# cfDNA deconvolution via NIPT of a pregnant woman after bone marrow transplant and donor egg IVF

**DOI:** 10.1186/s40246-021-00311-w

**Published:** 2021-02-23

**Authors:** Jianjiang Zhu, Feng Hui, Xuequn Mao, Shaoqin Zhang, Hong Qi, Yang Du

**Affiliations:** 1 Prenatal Diagnosis Center, Beijing Haidian Maternal and Child Health Hospital, Beijing, China; 2 Annoroad Gene Technology Co., Ltd., Beijing, China

**Keywords:** NIPT, Target sequencing, Fetal fraction, IVF, Transplant, Prenatal diagnostic

## Abstract

Cell-free DNA is known to be a mixture of DNA fragments originating from various tissue types and organs of the human body and can be utilized for several clinical applications and potentially more to be created. Non-invasive prenatal testing (NIPT), by high throughput sequencing of cell-free DNA (cfDNA), has been successfully applied in the clinical screening of fetal chromosomal aneuploidies, with more extended coverage under active research.

In this study, via a quite unique and rare NIPT sample, who has undergone both bone marrow transplant and donor egg IVF, we investigated the sources of oddness observed in the NIPT result using a combination of molecular genetics and genomic methods and eventually had the case fully resolved. Along the process, we devised a clinically viable process to dissect the sample mixture.

Eventually, we used the proposed scheme to evaluate the relatedness of individuals and the demultiplexed sample components following modified population genetics concepts, exemplifying a noninvasive prenatal paternity test prototype. For NIPT specific applicational concern, more thorough and detailed clinical information should therefore be collected prior to cfDNA-based screening procedure like NIPT and systematically reviewed when an abnormal report is obtained to improve genetic counseling and overall patient care.

## Introduction

Cell-free DNA (cfDNA) is known to be a mixture from several releasing sources organs [1], making it an opportunistic clinically biomarker for several non-invasive molecular applications. Cell-free fetal DNA (cffDNA) has been found in the plasma of pregnant women, originating from the placenta and enabling non-invasive prenatal testing of fetal chromosomal aneuploidies [2]. Most recently attempts have been made to extend the coverage to assess more comprehensive genomic alterations [3]. Non-invasive cancer assessment, more commonly known as liquid biopsy, has been enabled by the establishment of circulating tumor DNA (ctDNA) in the plasma of cancer patients [4]. Also in a more clinically critical field of application, organ transplantation, detection, and quantification of cfDNA of donor origin could be used to evaluate and monitor graft rejection [5]. In all these fields, the analytical outcome relies on the quantification of genetic differences of the unbalanced components dissected from the total cfDNA.

Noninvasive prenatal test (NIPT) using high throughput sequencing of maternal plasma cell-free DNA (cfDNA) to screen common fetal chromosomal aneuploidy has been widely applied in prenatal diagnosis worldwide. As one of the largest NIPT market and also being strictly regulated by IVD standard, NIPT in China has grown rapidly in recent years, with millions of pregnant women being tested every year. Many maternity hospitals in China have been enabled to operate independent clinical laboratory employing the latest NGS-based NIPT [6].

In this work, we looked into a unique NIPT case with several potentially interfering medical factors, rendering a complex genetic background of cfDNA. By combining populational polymorphic information, tissue-specific a priori, and sex chromosome dosage as biomarkers for cfDNA deconvolution, we devised a general framework to effectively dissect the cfDNA mixture and assess the genetic relatedness of the interleaved components.

## Material and method

### Sample information

An NIPT sample from a pregnant woman aged between 30 and 35 with initially normal medical history was received in 2019, at the gestation week of 16. The NIPT result was assessed to be of low risk for trisomy of chromosome 13/18/21. However, a large region of chromosome X showing a reduced depth of coverage, compared to normal pregnancy. On the other hand, the sample showed an estimated copy number ratio of 0.59/0.41 between chromosome X and chromosome Y, a chromosome Y-based estimate of the fetal fraction is 82%.

A prenatal diagnosis was later ordered to investigate the possible deletion of chromosome X. At the same time, a more detailed medical history of the patient was recovered. In 2008, being diagnosed with acute lymphoblastic leukemia (ALL), the woman underwent a bone marrow transplant donated by her relative. Moreover, the current pregnancy was also not conceived normally. After the transplantation and successful recovery, she was later diagnosed with premature ovarian failure (POF) due to extensive chemotherapy during the ALL treatment. The current gestation was fortunately made available after a successful donor egg IVF.

### Karyotyping analysis

White blood cells and oral mucosa cells were sampled from the pregnant woman and her husband during the consultation after the abnormal NIPT report, (Table 1), together with fetal cells collected from amniotic fluid. Standard karyotyping analysis and fluorescent in situ hybridization (FISH) were conducted to check the chromosome composition of selected samples.

### Capture sequencing panel design

In this study, a custom target region capture panel was designed to cover 739 chromosomal regions, which are all carefully selected with fewer repeats, consistent thermodynamic properties like GC content and nearest-neighbor melting temperature, and most importantly all carry populational polymorphic locus within the 30 bp of the 3’ end of the target region. Among these loci, 679 regions of variable length (average length of 100bp) are sparsely scattered on 22 autosomes, with a special focus of chromosome 13, 18, and 21, about 200 unique targets each (222 for chr13, 221 for chr18, and 194 for chr21, respectively); additionally, there are 30 unique regions each of ZFX and ZFY genes targeted to differentiate sex chromosomes.

### DNA preparation and sequencing

Five milliliters of peripheral venous blood from each individual was preserved and delivered to the laboratory in EDTA tubes (Sekisui, Tokyo, Japan) or Streck tubes (La Vista, NE, US). The plasma was separated after 2 rounds of centrifugation and stored at −80°C until DNA extraction. Cell-free DNA was extracted from plasma according to standard commercial protocols described in previous publications [7–9]. NIPT library was prepared using NMPA certified Kit (Registration No. 20173400331), and finally, 4.2 million single-end reads of 40bp were generated for each sample library using NextSeq 550AR (Annoroad Gene Tech., China).

On the other hand, for the capture sequencing run, 1 μg of the library mixture was hybridized with the SeqCap EZ Probes oligo pool (Roche Nimblegen, USA) using the target capture panel at 47 °C for 72 h followed by another round of PCR amplification in accordance with the standard procedure of the SeqCap EZ Probes handbook. The captured DNA fragments were purified using the Agencourt AMPure XP-nucleic acid purification kit and evaluated using Agilent 2100 Bioanalyzer (Agilent, USA) and quantitative PCR. Finally, the libraries were sequenced as PE150 using Illumina HiSeq X Ten system (Illumina, USA), with an average target sequencing depth of 2500X. All procedures were performed in a CAP-certified standard negative pressure laboratory (Beijing Annoroad Medical Laboratory, China) with constant temperature and humidity.

### Data analysis

The custom-made capture sequencing data analysis workflow was established to evaluate the genetic relatedness between individual samples. To eliminate low-quality reads, reads with more than 5% N or with at least 50% of all bases’ quality not larger than 30 were filtered out from the raw data with our in-house scripts, and sequencing adaptors were also removed. Using the Burrows-Wheeler alignment (BWA) tool [10], the remaining reads were aligned to human genome reference sequences (HG19, NCBI build 37) with default parameters. PCR duplications were further removed with SAMtools [11]. Germline mutation of gDNA samples was processed as the GATK best practice [12]. Plasma cfDNA samples were processed using a similar pipeline as previously described [13], in order to obtain the joint genotype calls of the fetal and maternal DNA.

Due to the relatively sparse distribution of the target loci on the panel and small sample size, it is not straightforward and reliable to obtain the phased haplotypes. We then introduce the match ratio statistics (mrs), which is defined as the number of identical genotypes of shared loci divided by the total number of common non-reference loci between a pair of samples, with the assumption that pair of individuals sharing the same genotype at the same loci are more likely to be duplicates or first-degree relatives, while all targeted loci are considered independent.

We also employed standard population genetics tools like PLINK [14] and KING [15] to infer the relatedness of the test samples. Due to the sparse loci distribution and small sample size, both tools provide rather uninformative kinship estimates, which are all zero. With “homog” mode of KING, we obtained the quantitative kinship estimates, which are not by the common definition of the kinship coefficients, but rather a pairwise similarity measure to infer the population structure.

## Result

The standard NIPT report of this particular patient is nothing out of the ordinary, with *Z*-scores of aneuploidies for chromosome 13/18/21 equal to 1.1626, − 0.2602, and − 0.46241, respectively. However, extremely high chromosome Y-based fetal fraction (~80%) and large-scale chromosome X depletion are observed when checking the data according to the expended NIPT scope, which includes Sex Chromosome Aneuploidies (SCA) and large sub-chromosomal copy number variants. According to the later clinical information update, the NIPT result is mostly explainable. The extremely high chromosome Y dosage of 82% male DNA in the plasma compared to normal pregnancy are assumed largely contributed by the male donor blood white cells with possible addition of a male fetus, while 18% are female cfDNA which most likely originated from the mother also with the possible addition of a female fetus. Considering such medical history, it is thus impossible to infer fetal gender and fetal fraction directly via Y chromosome dosage. A seqFF [16]-based in-house prediction method gives a 9.36% estimation of the fetal fraction, inferring cfDNA of placenta origin. It is therefore the plasma cfDNA could be a mixture of 3 different sources: A, 46,XY donor-maternal plasma cfDNA; B, 46,XX not replaced maternal cfDNA; C, fetal/placenta cfDNA of unknown gender.

**Fig. 1:**
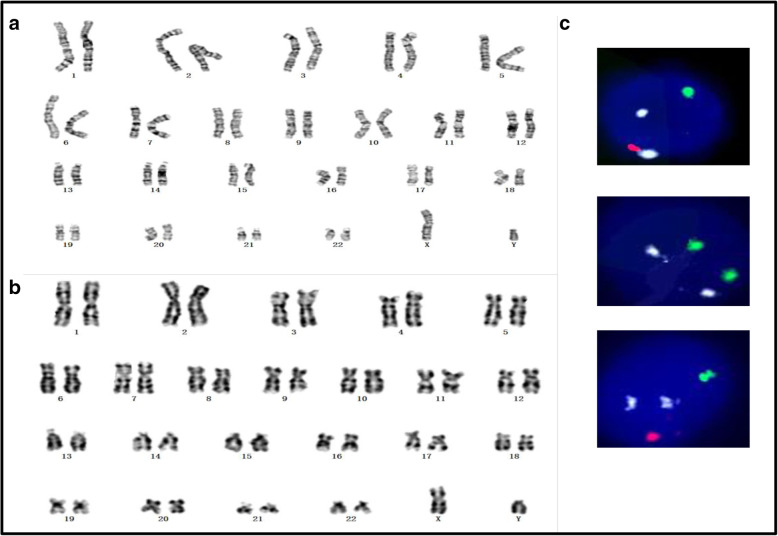
G-banding karyotype of samples of the pregnant woman (**a**) and amniocytes of the fetus (**b**). Interphase FISH analysis of the pregnancy, peripheral blood cells (C1, top), oral mucosa cells (C2, middle), and uncultured amniocytes of the fetus (C3, bottom) with chromosome 18 (aqua), X (green), and Y (red)

G-banding karyotype of blood sample from the pregnant woman (Fig. 1a) and amniocytes of the fetus (Fig. 1b) all suggest normal karyotype of a 46,XY male. Interphase FISH analysis of the pregnancy, peripheral blood cells (Fig. 1C1, top), oral mucosa cells (Fig. 1C2, middle), and uncultured amniocytes of the fetus (Fig. 1C3, bottom) show discrepant findings, with the peripheral blood cells almost completely replaced with male chromosomes, whereas the oral mucosa cells are still dominantly composed of female chromosomes. Both analyses conclude the fetus is a normal 46,XY male.

**Table 1:** Sample information. *Gender of the test sample is predicted with the sequencing data

Sample ID	Role	Sample type	DNA type	Sample gender^*^
W0120237003	Mother	White blood cell	gDNA	Male
W0120237004	Mother	Plasma	cfDNA	Female
W0120237005	Mother	Oral mucosa	gDNA	Female
W0120237006	Father	White blood cell	gDNA	Male
W0120237007	Father	Plasma	cfDNA	Male
W0120237008	Fetus	Amniotic fluid	gDNA	Male

**Table 2:** Relatedness of different types of pairwise relationship. “Kinship” was produced with PLINK, and Kinship-homog was calculated with “homog” mode of KING. “mrs” was defined as the number of identical genotypes of shared loci divided by the total number of common non-reference loci between a pair of samples. “Type” is defined as “MZD,” replicated of the same biological sample; “1st” and “NR” indicate a similar biological relationship between first-degree relationship as a sibling or no genetic relationship. The suffix of “major” and “minor” indicate the components of plasma sample after deconvolution

Lib-Pair	mrs	Kinship	Kinship-homog	Type
W0120237005-W0120237004major	0.951219512	0	−0.3474	MZD
W0120237006-W0120237007	0.907407407	0	−0.271	MZD
W0120237003-W0120237005	0.902439024	0	−0.0113	MZD/1st
W0120237007-W0120237004minor	0.777777778	0	−0.9107	1st
W0120237003-W0120237004major	0.755102041	0	−0.4752	1st
W0120237005-W0120237004minor	0.707317073	0	−0.719	NR
W0120237004major-W0120237004minor	0.693877551	0	−0.3071	NR
W0120237006-W0120237008	0.690909091	0	−0.0746	1st
W0120237007-W0120237008	0.685185185	0	−0.6575	1st
W0120237007-W0120237004major	0.666666667	0	−1.1214	NR
W0120237005-W0120237008	0.585365854	0	−0.4468	NR
W0120237006-W0120237004major	0.571428571	0	−0.6758	NR
W0120237008-W0120237004minor	0.563106796	0	−0.6385	MZD/NR
W0120237006-W0120237004minor	0.563106796	0	−0.5314	1st/NR
W0120237005-W0120237006	0.56097561	0	−0.2095	NR
W0120237008-W0120237004major	0.540816327	0	−0.8303	NR
W0120237003-W0120237007	0.518518519	0	−0.8658	NR
W0120237005-W0120237007	0.512195122	0	−0.7805	NR
W0120237003-W0120237006	0.509090909	0	−0.2	NR
W0120237003-W0120237008	0.473333333	0	−0.3805	NR
W0120237003-W0120237004minor	0.436893204	0	−0.8847	1st/NR

**Fig. 2:**
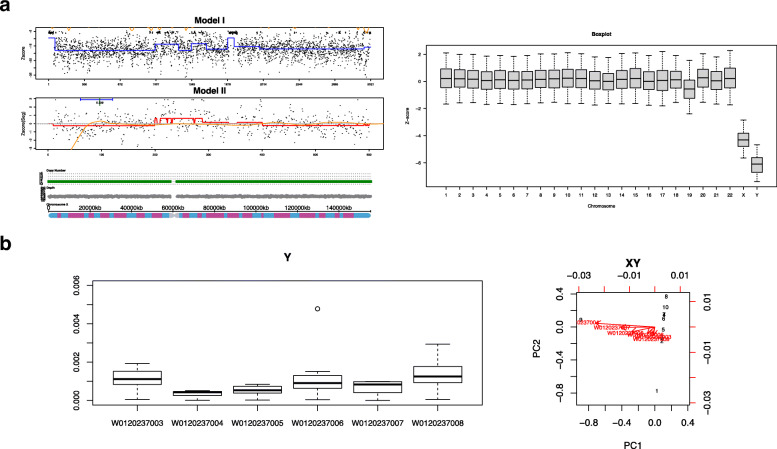
**a** (left) Normalized NIPT depth profile of chromosome X showing significantly reduced coverage comparing to a background of normal fetus, (right) boxplot of relative normalized depth profiles across chromosomes; **b** Normalized depth coverage of chromosome Y regions across samples using panel data (left), with a PCA plots showing the overall similarity of the samples (right)

In Fig. 2a (left), the normalized depth profile of chromosome X is shown, significantly reduced coverage of the X chromosome is seen, representing a copy number of approximately 1, which is consistent with the previous karyotype result of blood cells. Boxplot of relative dosage between sex chromosomes of the NIPT data provide further evidence of a similar ratio between chromosome Y and chromosome X, Fig. 2a (right), which is close to a male sample with equivalent copy dosage estimates yet with slightly more female DNA.

Regarding the gender of the test samples, since the mother underwent a bone marrow transplant donated by a close male relative (first degree) in 2008, it would be interesting to check for the gender differences in the obtained gDNA and cfDNA samples from the mother. In Fig. 2, normalized read depth of uniquely mapping reads to target regions of chromosome Y suggests that maternal plasma (W0120237004) and gDNA sample from the oral swab (W0120237005) contains a lower percentage of DNA molecules from the Y chromosome, thus more likely to be female, this is consistent with our hypotheses, that 46,XY cells from the male donor gradually replace the maternal white blood cells making sample profile of W0120237003 more similar to a male. Another source of male DNA is from the 46,XY fetus which were at the moment about 16w of gestation. And finally, the amniocentesis report came back as a normal 46,XY fetus. On top of this information, we could reliably estimate the percentage of the 3 different biological sources A (46,XY, 72.64%), B (46,XX, 18%), and C (46,XY, 9.36%) in the tested cfDNA NIPT sample.

**Fig. 3:**
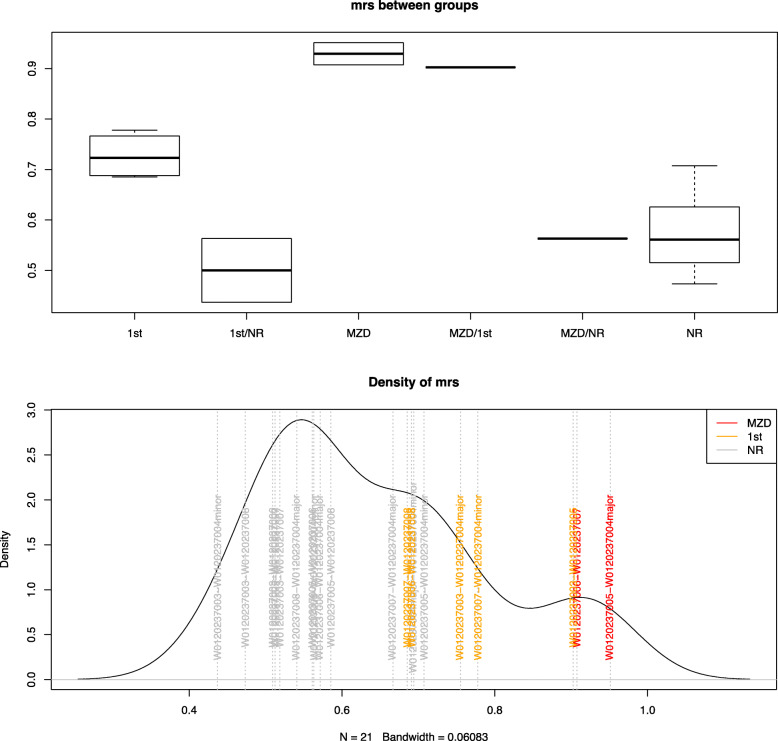
Boxplot of the estimated relatedness of different types of pairwise relationship

In Fig. 3, our proposed matching ratio statistics (mrs) shows rather informative group segregation with respect to their biological relationships ranking, with “MZD” suggesting a self-duplication or a monozygotic twin, “1st” representing first-degree relatives, “NR” indicating no biological relationship is assumed between the pair. For some of the pairs, we are not confident to assign a group flag, since either flag can exist or a mixture of the two conditions rendering it not exactly belonging to such a mutually exclusive grouping scheme.

**Fig. 4:**
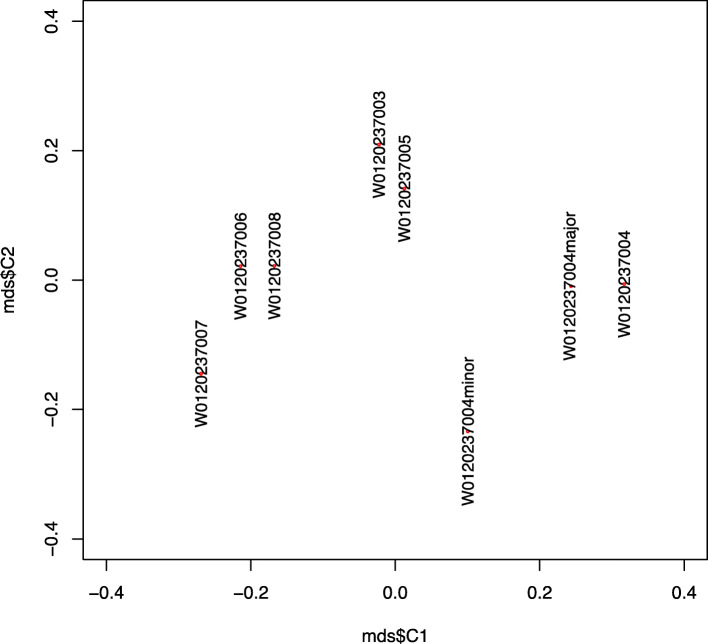
Two-dimensional MDS plot of genetic distances between samples using PLINK

**Fig. 5:**
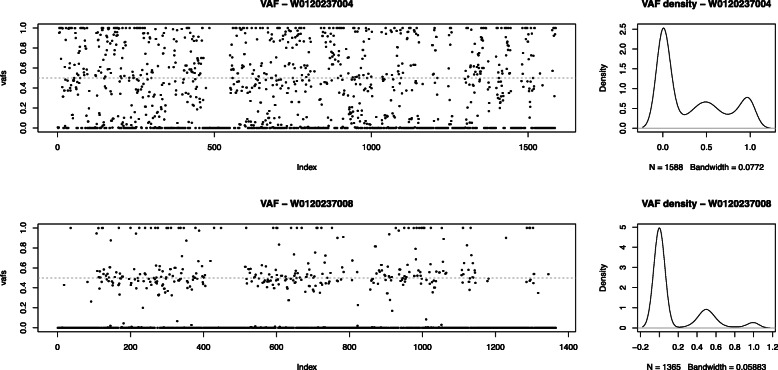
Comparing to a typical gDNA sample W0120237008 (bottom) of the fetus, minor allele frequency of the maternal plasma sample W0120237004 (top), suggesting a mixture of DNA from several biological individuals

Though the mostly negative kinship (homog) estimates reported are not meaningful by the original definition, (Table 2), in which 0.354 and 0.177 are considered boundaries for biological duplicates and 1st-degree relationships, respectively [15]. Genotype data were used to illustrate their genetic distances using PLINK. A two-dimensional MDS plot is shown in Fig. 4 with the first two major components, from which we can see biologically related samples are placed more closely, whereas gDNA sample W0120237006 with 1st-degree relationship like W0120237008 are somehow closer comparing to the distance with its cfDNA “duplicate” W0120237007. A similar pattern exists for the maternal cfDNA sample W0120237004, yet with a more complex genetic background. Normally homozygous and heterozygous loci in a single gDNA sample should present three horizontal bands in the distribution of MAF, as in Fig. 5, the less exogenous DNA it contains the more these three bands will be centered at 0, 0.5, and 1. Here, we could observe a quite sparse distribution of the allele frequency in W0120237004, suggesting rather dynamic mixture of more than 2 different sources of the genetic background of unknown proportion.

## Discussion

In this study, we accidentally encountered a rare prenatal diagnostic case, which combines several low-frequency clinical events and creates a rather complex genetic background for NIPT. Using several molecular genetics and genomic tools, we investigated the source of abnormal NIPT result and illustrated genetic relatedness and composition of the dissected component.

It is worth noticing that in future prenatal diagnostic consultation, before conducting NIPT, relevant clinical information must be thoroughly collected. After obtaining abnormal NIPT report, specific evidence other than normal reporting statistics for the aneuploidy inference should be collected to facilitate a precise and satisfactory consultation. Specially in the case of organ transplantation prior to NIPT, a male donor must be identified, which might cause inference error in fetal fraction estimation and also sample quality. However, a female donor would most likely have no impact over the analytical process as long as sufficient cffDNA exists in the blood sample, this must be reliably inferred via analytical methods, like the ones we employed in this study. In the case of low-frequency mosaicism events (like copy number variants and chromosomal aneuploidy) that exist in the donor cells or occurred during cell preliberation, the resulting abnormal coverage statistics might be erroneously inferred as a false positive event carried by the fetus. In such a case, maternal background profile must be ruled out when reporting a positive result, using a similar strategy as in somatic variant calling in circulating tumor DNA (ctDNA) analysis.

Unfortunately, we did not obtain any biological sample from the bone-marrow-donor relative of the mother and also the egg donor. Otherwise, we can devise a more unified decomposition model using polymorphic loci and all parental donors’ genotype profiles involved in the mixture, extending the pseudo-tetraploid genotyping (PTG) methodology to pseudo-hexaploid genotyping by incorporating additional donor fraction coefficient into the model and solving a similar maximum likelihood model [13].

On the other hand, with such a mixed genetic background, our customized SNP-based panel which utilizes populational polymorphism to quantify genetic difference can correctly infer the relative genetic similarity between samples and provide a classification of the degree of relationship. It can also be utilized to distinguish zygosity in twin pregnancies noninvasively [17]. Similar to the existing Non-Invasive Prenatal Paternity Test (NIPPT) prototype [18], ultimately the proposed protocol enables yet another streamlined NIPPT setting using panel-based NIPT in the near future, which already combines the utilities of chromosomal aneuploidy detection, CNV detection and also single-gene disorders identification [3]. Such a unified solution could greatly improve prenatal diagnostic yield and further elucidate understanding of complications in maternal-fetal medicine.

## Data Availability

We are not able to release and share the raw sequencing data and more detailed patient information given specific restric under local regulatory rules regarding human genetic material. Processed data are individually available upon request.
